# Epidemiological, clinical and laboratory features of acute Q fever in a cohort of hospitalized patients in a regional hospital, Israel, 2012-2018

**DOI:** 10.1371/journal.pntd.0009573

**Published:** 2021-07-15

**Authors:** Talya Finn, Frida Babushkin, Keren Geller, Hanna Alexander, Svetlana Paikin, Jonathan Lellouche, Yafit Atiya-Nasagi, Regev Cohen

**Affiliations:** 1 Infectious Diseases Department, Sanz Medical Center, Laniado Hospital, Netanya, Israel; 2 The Ruth and Bruce Rappaport Faculty of Medicine, Technion, Haifa, Israel; 3 Microbiology Laboratory, Sanz Medical Center, Laniado Hospital, Netanya, Israel; 4 Department of Biochemistry and Molecular Genetics, Israel Institute for Biological Research, Ness Ziona, Israel; University of Texas at San Antonio John Peace Library, UNITED STATES

## Abstract

**Introduction:**

Acute Q fever is endemic in Israel, yet the clinical and laboratory picture is poorly defined.

**Methods:**

A retrospective study reviewing the medical records of acute Q fever patients, conducted in a single hospital in the Sharon district, Israel. Serum samples from suspected cases were preliminary tested by a qualitative enzyme immunoassay (EIA). Confirmatory testing at the reference laboratory used an indirect immunofluorescence assay (IFA). Positive cases were defined as fever with at least one other symptom and accepted laboratory criteria such as a single-phase II immunoglobulin G (IgG) antibody titer ≥1:200. Cases not fulfilling these criteria and in which acute Q fever was excluded, served as a control group.

**Results:**

Between January 2012 and May 2018, 484 patients tested positive. After confirmatory testing, 65 (13.4%) were positive for acute Q fever (with requisite clinical picture), 171 (35.3%) were definitely not infected, the remaining 248 were excluded because of past/chronic/undetermined infection. The average age was 58 years and 66% were males. Most resided in urban areas with rare animal exposure. Pneumonia was seen in 57% of cases and a combination with headache/hepatitis was highly suggestive of acute Q fever diagnosis. Syncope/presyncope, fall and arthritis were more common in acute Q fever cases. Laboratory indexes were similar to the control group, except for erythrocyte sedimentation rate (ESR) which was more common and higher in the study group.

**Conclusion:**

Acute Q fever in the Sharon district could be better diagnosed by using a syndromic approach in combination with improved rapid diagnostic testing.

## Author summary

Acute Q fever is endemic in Israel, yet the clinical and laboratory picture is poorly defined. We performed a retrospective study reviewing medical records of acute Q fever patients compared to those for whom acute Q fever was excluded. Serum samples from suspected cases were preliminary tested by a qualitative enzyme immunoassay (EIA). Confirmatory testing at the reference laboratory used an indirect immunofluorescence assay (IFA). Positive cases were defined as fever with at least one other symptom and accepted laboratory criteria. Cases not fulfilling these criteria and in which acute Q fever was excluded, served as a control group. Over 6 years, 484 patients tested positive by EIA. After confirmatory testing, 65 (13.4%) were positive for acute Q fever (with requisite clinical picture), 171 (35.3%) were definitely not infected, the remaining 248 were excluded because of past/chronic/undetermined infection. Most resided in urban areas without animal exposure. Pneumonia was seen in 57% of cases and a combination with headache/hepatitis was highly suggestive of acute Q fever diagnosis. Syncope/presyncope, fall and arthritis were more common in acute Q fever cases. We conclude that a syndromic approach to acute Q fever in combination with improved rapid diagnostic testing would be beneficial.

## Introduction

Q fever is a ubiquitous zoonotic disease caused by an obligate intracellular bacterium, *Coxiella burnetii*. Acute infection has a wide array of presentations, from asymptomatic disease or mild respiratory symptoms ("flu-like" illness) to severe syndromes including atypical pneumonia, hepatitis, encephalitis and other manifestations[1]. Probably the most common manifestation is one of a self-limited febrile illness, possibly with an incidental consolidation on chest radiograph[2]. There have been reports of severe headache in as many as 75% of those with Q fever pneumonia[3]. Chronic presentations of Q fever can be endocarditis or other endovascular infection, osteomyelitis and in rare cases, pericardial effusions[1].

Acute Q fever may have different clinical presentations depending, among other factors, on the geographic location and the reporting rates. According to the Centers of Disease Control and Prevention (CDC), the disease is considered endemic in the Middle East[4]. The largest known outbreak reported in recent years involved approximately 4,000 cases between 2007–2010 in the Netherlands, caused by exposure to infected goat milk, meat, and manure[5].

Q fever transmission may result from inhalation of infected aerosols originating from multiple animal lineages including birds, rodents, livestock, and cats as well as from exposure to ticks or contaminated milk[6]. In addition, there have been rare cases of human to human transmission via measures such as blood transfusions or during obstetric deliveries[7].

There seems to be seasonal variation in some areas of the world, relating to rainfall and wind speeds, for example around lambing season in France[8]. A study investigating the impact of climate and demographics on the incidence of disease concluded that airborne transmission is the most powerful predictor of human Q fever incidence rates[9].

Laboratory abnormalities can be minimal and non-specific and include mildly abnormal transaminases, thrombocytopenia/thrombocytosis and typically the absence of leukocytosis[10]. The gold standard test for Q fever diagnosis is serology using an indirect immunofluorescence assay (IFA)[4]. Antibodies usually appear 7–10 days after the onset of the disease. Molecular methods, based on real-time polymerase chain reaction (PCR), are also used for early diagnosis of acute Q fever[11].

Although Q fever is a notifiable disease in Israel, Ministry of Health records from 1951–2010 show that very few cases are reported[12] despite the general knowledge of Q fever endemicity. In one report from 2004, the prevalence was 1–2 cases per 100,000 people[13]. There have been several published studies and outbreak reports of Q fever from Israel in the last decade[14–17]. A recent report from the Sharon district regarding 38 hospitalized patients with acute Q fever found that they presented as a non-specific febrile disease and pneumonia in 45% of cases and had no predilection for rural habitation. In this study, common laboratory features were hepatocellular enzyme disturbances and thrombocytopenia[18]. These findings are in contrast with an earlier report from Israel[16].

We suspect that the Israeli description of this disease, regarding both the clinical and laboratory features, is insufficient, leading to frequent misdiagnosis and missed treatment opportunities. IFA is not widely accessible out of the reference laboratory for Rickettsial diseases in Israel. Hence, local laboratories use other, less specific, immunoassays and suspected samples are sent for confirmatory IFA in the reference laboratory. High rates of false positive test results (using non IFA testing) lead to difficulties managing suspected acute Q fever cases. We conducted a retrospective analysis of acute Q fever cases diagnosed in our facility and compared their clinical and laboratory characteristics to a group of patients for whom acute Q fever was excluded. The aim was to see if we could build a clearer picture of how acute Q fever presents in Israel and improve the positive predictive power of the non-IFA testing.

## Methods

### Ethics statement

This study was approved by the local institutional review board of Sanz Medical Center, Laniado hospital (0028-18-LND).

### Setting

The study was conducted in Sanz Medical Center, a 400 bed-university affiliated hospital located in the city of Netanya, Sharon district. The hospital serves a population of ~250,000 located in a variety of local rural and urban areas.

### Patient and laboratory data

This was a retrospective, comparative study, reviewing the medical records of all patients who tested positive for *C*. *burnetii* using an enzyme immunoassay (EIA) (ImmunoDOT, GenBio, San Diego, CA, catalogue number: 4050), performed in our institution, between January 2012-May 2018. ImmunoDOT uses EIA dot technique for the detection of IgG and IgM antibodies to *C*.*burnetti*[19]. Results can be positive, negative or equivocal. All inconclusive or positive samples were stored at 4°C and a batch sent every six days for IFA confirmation to the Israel Institute for Biological Research (IIBR), the national reference laboratory for Rickettsial diseases in Israel. Medical records were reviewed by two infectious diseases physicians.

Acute Q fever was diagnosed using clinical and laboratory criteria: fever with at least one other symptom (pneumonia, headache, myalgia, hepatitis, meningoencephalitis) and a single phase II immunoglobulin G (IgG) antibody titer ≥1:200 with or without positive immunoglobulin M (IgM) antibody titer, or a fourfold increase or seroconversion in phase II IgG antibody titers as measured by IFA between paired samples. Chronic Q fever cases were excluded as well as those who did not meet clinical and laboratory criterion or for whom an alternative diagnosis was made such as bacteremia. Demographic, clinical, laboratory and radiological details were recorded.

### Statistical analysis

Patient characteristics were summarized using descriptive statistics. Differences between categorical and continuous variables were calculated using Fisher’s exact test, Student’s t-test and Mann-Whitney tests, as appropriate. All statistical analyses were performed using GraphPad Prism 7. In all statistical analyses, a two-sided p-value less than 0.05 was considered statistically significant.

## Results

### Acute Q fever cases

**Fig 1:**
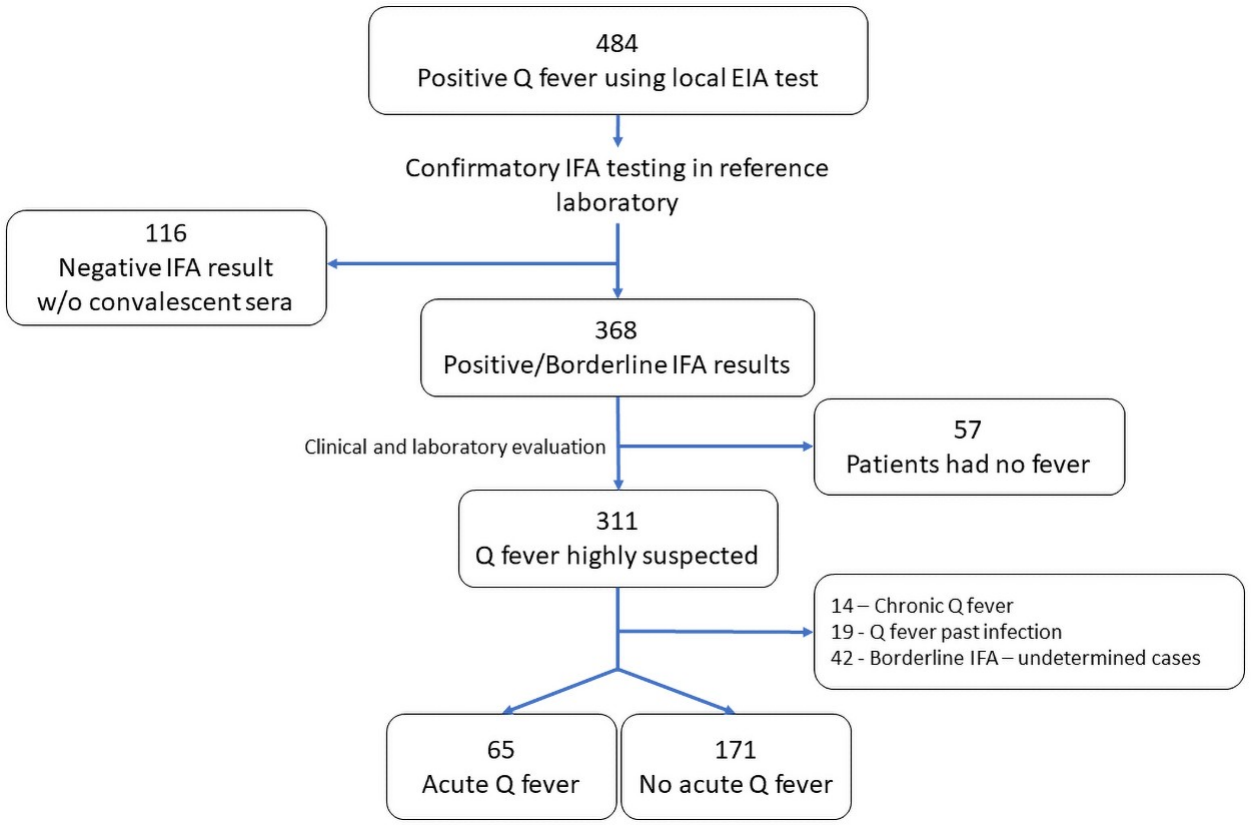
Explanation regarding inclusion and exclusion of cases.

Between January 2012 and May 2018, a total of 484 tests were inconclusive or positive for *C*. *burnetii* using in-house EIA testing. All of these tests were sent for confirmatory IFA testing and after reviewing the medical notes along with the serology results, we classified 65 (13.4%) as acute Q fever and 171 (35.4%) as definitely negative cases. The remaining 248 (51.2%) negative cases were excluded (Fig 1). Of the 65 acute Q fever cases, 23 (35%) cases had repeat IFA tests.

### Patient characteristics

**Fig 2:**
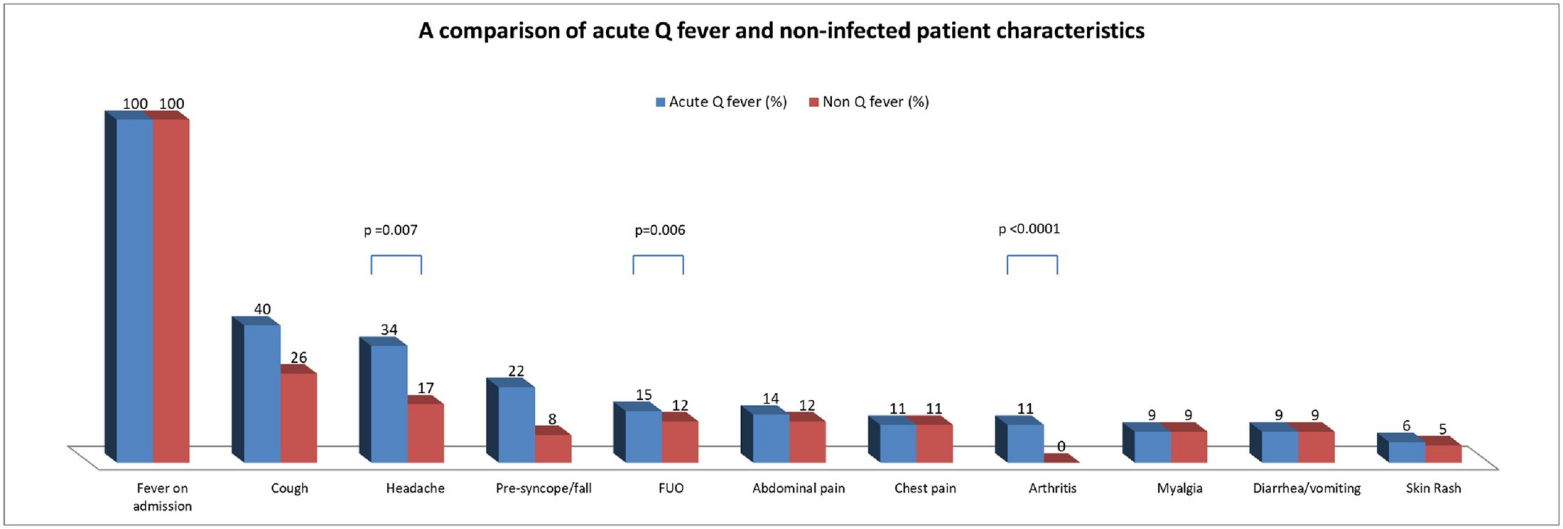
Bar graph demonstrating the percentage of patients in the acute Q fever versus non infected group with each clinical characteristic. In the case of a significant difference the p value is written above.

Demographics and medical history of the acute Q fever group were compared to the non-Q fever group (Table 1 and Fig 2). The average age was 58 and 59 years, respectively. Male predominance was evident in both groups (66% and 63% respectively). Rural residency was not a risk factor for disease. Definite animal exposure was reported in 22% of the acute Q fever group and 15% of the non-Q fever group (p = NS). History of this exposure was obtained in only 40% of cases.

**Table 1:** Demographic and clinical characteristics of acute Q fever cases as compared with non-Q fever cases.

		Acute Q fever n = 65	Non-Q fever n = 171	Fisher exact test: p value, Relative risk (95% confidence interval)
Age, years, Mean (range)		58 (20–98)	59 (17–95)	
Male gender (%)		43 (66)	107 (63)	
Urban residence (%)		56 (86)	160 (93)	
Nursing home residence (%)		3 (5)	8 (5)	
Animal exposure	Reported (%)	14 (22)	25 (15)	
	Not reported (%)	12 (18)	45 (26)	
	Undocumented (%)	39 (60)	101 (59)	
Pregnancy (%)		2 (3)	4 (2)	
**Past medical history**				
Prosthetic valve (%)		5 (8)	15 (9)	
Permanent pacemaker (%)		3 (5)	7 (4)	
Any immunosuppression (%)		9 (13)	22 (13)	
Post splenectomy (%)		2 (3)	2 (1)	
Non Hodgkin’s lymphoma (%)		2 (3)	7 (4)	
Solid organ transplantation (%)		1 (1)	1 (0.5)	
Current corticosteroid therapy (%)		4 (6)	5 (3)	
Current immunomodulation therapy* (%)		2 (3)	2 (1)	
Current malignancy (%)		3 (5)	8 (5)	
Current chemotherapy (%)		2 (3)	1 (0.5)	
**Symptoms and signs**				
Fever on admission (%)		65 (100)	171 (100)	
Cough (%)		26 (40)	45 (26)	p = 0.055**
Headache (%)		22 (34)	29 (17)	p = 0.007, RR = 1.99 (1.2–3.1)
Pre-syncope/Syncope/Fall (%)		14 (22)	14 (8)	p = 0.006, RR = 2.63 (1.3–5.1)
Fever of unknown origin (%)		10 (15)	21 (12)	
Abdominal pain (%)		9 (14)	20 (12)	
Chest pain (%)		7 (11)	19 (11)	
Arthritis (%)		7 (11)	0	p<0.0001, RR = ∞ (2-∞)
Myalgia (%)		6 (9)	16 (9)	
Diarrhea/vomiting (%)		6 (9)	16 (9)	
Skin rash (%)		4 (6)	8 (5)	
**Diagnoses**				
CAP		37 (57)	66 (39)	p = 0.012, RR = 1.47 (1.09–1.94)
Bilateral CAP (%)		10 (15)	22 (13)	
CAP accompanied with cough (%)		21 (32)	32 (18)	p = 0.03, RR = 1.72 (1.07–2.7)
CAP accompanied with headache (%)		8 (12)	4 (2)	p = 0.004, RR = 5.2 (1.7–15.9)
Liver function abnormalities (%)		18 (28)	66 (39)	
CAP accompanied with liver enzymes abnormality (%)		13 (20)	21 (12)	p = 0.14
CAP accompanied with headache and liver enzymes abnormality (%)		4 (6)	2 (1)	p = 0.05, RR = 5.2 (1.1–24)
CAP accompanied with thrombocytopenia (%)		12 (18)	16 (9)	p = 0.07**
Encephalitis (%)		3 (5)	9 (5)	
Headache accompanied with liver enzymes abnormality (%)		7 (11)	15 (9)	

*Cyclosporin A, anti TNF alpha,

**non-significant.

CAP—community acquired pneumonia

### Clinical characteristics

**Fig 3:**
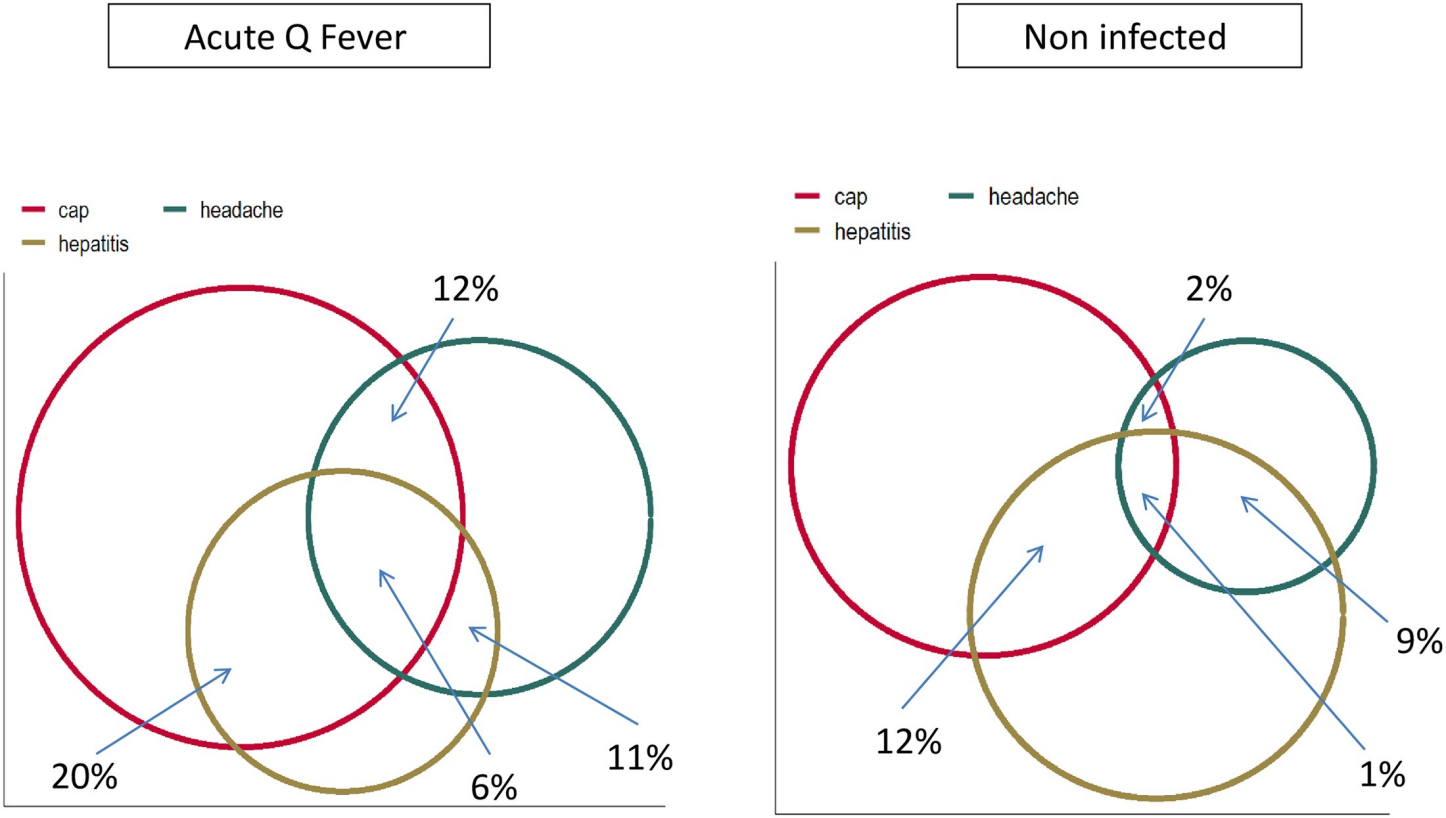
Venn diagram showing the percentage of patients with each “syndromic picture” in the acute Q fever and non infected group. Cap—community acquired pneumonia.

The most common clinical presentation of acute Q fever was of community acquired pneumonia (CAP), seen in 37/65 (57%) of cases (compared with 39% in the control group), p = 0.012, relative risk (RR) = 1.47, confidence interval (CI) = 1.09–1.94. CAP accompanied with headache was evident in 8/65 (12%) of cases and in only 2% of the control group (p = 0.004, RR = 5.2, CI = 1.7–15.9). CAP accompanied with liver enzyme abnormalities occurred in 20% vs 12% (p = 0.14), and CAP accompanied with elevation of liver enzymes as well as headache, in 6% vs 1% (p = 0.05, RR = 5.2 (1.1–24). CAP was more commonly symptomatic (reported cough) in the acute Q fever group (32% vs 18%, p = 0.03). The incidence of CAP with thrombocytopenia was similar in the acute Q fever versus the non infected group (p = 0.07). These key findings are presented in Table 1 and the Venn Diagram in Fig 3.

Regarding acute Q fever symptoms—cough was the most common symptom (40%), followed by headache (34%) and syncope/presyncope/fall at admission (22%). Both headache and syncope were significantly more common (RR = 1.99 and 2.6, respectively). Fever of unknown origin (FUO) was the presenting feature in 15% of cases. Arthritis was seen in 7 cases (11%) and was absent in the non-Q fever group (p<0.0001). Encephalitis was documented in 5%. More data are depicted in Table 1 and Fig 2.

### Laboratory features

During the first 3 days of hospitalization, patients with acute Q fever had hepatocellular or cholestatic enzymes elevation in 14–34% and mild hyperbilirubinemia in 11%, with no significant difference when compared to the non-Q fever group. In 51% of cases the leukocyte count was abnormal, leukocytosis more common than leukopenia (43% vs 8%), and thrombocytopenia was evident in 26%. C-reactive protein was elevated in 92% of cases during the first 3 days of hospitalization, averaged at 110mg/dL. ESR was elevated in 83% of cases vs 67% in the control group (p = 0.04), and was higher (mean 54mm/hour vs 43mm/hour, p = 0.06). More results are shown in Table 2.

**Table 2:** Laboratory, imaging, and outcomes of acute Q fever cases as compared with non-Q fever cases.

	Acute Q fever n = 65	Non-Q fever n = 171	Student’s t test
**Laboratory results in the first 3 days of hospitalization**			
**Liver indexes**			
Abnormal ALT (>41 IU/L) (%)	15/55 (27)	49/159 (31)	NS
Mean ALT (IU/L)	155	286	NS
Abnormal AST (>40 IU/L) (%)	22/64 (34)	70/169 (41)	NS
Mean AST (IU/L)	136	276	NS
Abnormal ALP (>130 IU/L) (%)	9/64 (14)	35/169 (21)	NS
Mean ALP (IU/L)	163	201	NS
Abnormal GGT (>61 IU/L) (%)	17/55 (31)	52/156 (33)	NS
Mean GGT (IU/L)	185	185	NS
Abnormal Bilirubin (>1.4 mg/dL) (%)	7/63 (11)	37/169 (22)	NS
Mean Bilirubin (mg/dL)	2.24	2.14	NS
**Cellular blood components**			
Leukocytosis (>11,000 cells/mcl) (%)	28/65 (43)	66/171 (39)	NS
Mean leukocytosis value (cells/mcl)	17,678	25,918	NS
Leukopenia (<4,000 cells/mcl) (%)	5/65 (8)	28/171 (16)	NS
Mean leukopenia value (cells/mcl)	3,534	2,461	NS
Thrombocytopenia (>400,000 cells/mcl) (%)	17/65 (26)	64/171 (37)	NS
Mean thrombocytopenia value (cells/mcl)	103,000	102,000	NS
**Inflammation indexes**			
Elevated CRP (>5mg/dL) (%)	47/51 (92)	110/132 (83)	NS
Mean CRP (mg/dL)	110	122	NS
Elevated ESR (%)	44/53 (83)	82/122 (67)	p = 0.04
Mean ESR (mm/hour)	54	43	p = 0.06
**Muscle indexes**			
Abnormal CK (>190 IU/L)	19/60 (32)	49/151 (32)	NS
Mean CK (IU/L)	413	1264	NS
**Echocardiography**			
TTE performed (%)	23/65 (35)	80/171 (47)	
Valvulopathy found in TTE (%)	4/23 (17)	25/80 (31)	
Vegetation found in TTE study (%)	1/23 (4)	4/80 (5)	
TEE performed (%)	3/23 (13)	7/80 (8)	
Vegetation found in TEE study (%)	1/23 (4)	1/80 (1)	
**Outcomes**			
ICU admission	6/65 (9)	26/171 (15)	
Death during hospitalization	3/65 (5)	17/171 (10)	

ALT—Alanine aminotransferase, AST—Aspartate transaminase, ALP—Alkaline phosphatase, GGT—Gamma glutamyl transpeptidase, CRP—C-reactive protein, ESR—Erythrocyte sedimentation rate, CK—Creatine kinase, TTE—trans-thoracic echocardiography, TEE—trans-esophageal echocardiography, ICU—Intensive care unit, NS—non significant. Valvulopathy- not including mild valvular changes

### Outcomes

Average time from symptom onset to admission was 18.9 days in the acute Q fever group, and 7.8 days in the control group (p = 0.13). Mean length of hospitalization was 8.9 days (range 1–81) and 13.6 days (0–369), respectively (p = 0.2).

Of the acute Q fever cases, 6 (9%) were admitted to the intensive care unit (ICU) and 27 (15%) in the non-Q fever group. Three (5%) acute Q fever cases died during hospitalization vs 17 (10%) in the non-infected group. Upon discharge, recommendations to repeat serological testing were made in 46 of all the cases, 29/61 (48%) of the acute Q fever group and 17/149 (11%) of the non-infected cases (excluding those who died during hospitalization). Forty-two (65%) of the infected group were given doxycycline versus 79 (46%) of the non-infected group.

### Seasonality

Although 23/65 (35%) of acute Q fever cases were diagnosed between April and June, as opposed to 18–24% during each of the other year’s quarters, there was no obvious seasonality.

## Discussion

The clinical and laboratory presentation of acute Q fever in Israel is still poorly described and two studies conducted a decade apart described somewhat different clinical and laboratory features[16,18]. Both reported febrile disease as the main presentation, and CAP was reported in 30–45% of cases. Differences were seen regarding the rates of reported headache, hepatitis and thrombocytopenia. In this study, we report higher CAP rates (57% of cases) than both previous studies. A third of our cases had headache as opposed to the recent study which reported only 1% with this symptom[18]. We also found that leukocytosis was much more common than leukopenia (43% and 8%, respectively). The presence of hepatitis with only fever, although found in about third of the cases, was not different as compared to our control group.

We focused on the co-occurrence of CAP with other common features of acute Q fever, namely severe headache and hepatitis, as clues to the diagnosis. We found that the presence of CAP with headache or CAP with both headache and hepatitis increases the probability of a positive diagnosis 5-fold. Aside from headache, other neurological symptoms such as syncope/presyncope or falls, and the co-occurrence of arthritis also hint at the diagnosis. Consideration of these specific combinations of clinical presentations as triggers for clinical suspicion may facilitate timely diagnosis of Q fever patients hospitalized with CAP. A possible explanation for the discrepancies between previous studies may be in part due to variable geographic location. Ergas *et al*[16] collected cases from the IIBR database, representing all parts of Israel, whilst our study and the study conducted by Reisfeld *et al*[18] described patients living in the Sharon district only. The impact of geographic location upon the different clinical picture is well known in acute Q fever[1,20–23]. These variations have been described even within the same country as noted in Spain where pneumonia is more common in the Basque region[21] and hepatitis more common in Andalusia[24].

By definition, all cases in the current study had fever—the most common sign of acute Q fever[1]. In 15% of our cases, FUO was diagnosed before acute Q fever was evident. Prolonged fever is a common finding in acute Q fever, described previously from Israel[16]. Hence, FUO in hospitalized patients should be investigated for Q fever.

Epidemiological features of acute Q fever in our cohort concurred with other reports: average age of 58 years, male predominance and usually occurring among healthy patients[25]. Urban residency was the rule (55/65 positive cases), often with no animal exposure. There are several hypotheses about the Q fever reservoir in Israel, including cattle as well as the plentitude of domestic animals and stray cats in urban areas. Amitai *et al* described an outbreak amongst 144/322 students at a boarding school in an urban area in central Israel, the cause of which was thought to have been stray cats contaminating a central air conditioning unit[17]. Attempts to isolate *C*. *burnetii* from the fleas of stray cats were unsuccessful[26].

Regarding the laboratory results of our study, only the ESR (erythrocyte sedimentation rate) was higher and more commonly seen in the acute Q fever group than the control group. Similar results were previously reported from Israel[16] and elsewhere[27,28]. All other indexes were similar.

In this study, we screened suspected patients with an EIA based on dot immunoblotting. Although Cowley and colleagues demonstrated that dot immunoblotting was as sensitive and as specific as IFA[29], we found a poor correlation between these two methods. The interpretation of dot immunoblotting involves interpreting the number of dots that are "positive" and is therefore "operator-dependent". The manufacturing instructions discuss equivocal results and even recommend possibly interpreting them as negative[19]. IFA also has its limitations. Interpretation of IFA results often involves taking into account the clinical picture because the test cannot differentiate between past or present infection nor can it detect cases before seroconversion.

Testing in the reference laboratory can take weeks and patients are frequently discharged before the results return. Inadequate "in house" laboratory test results have a variety of effects. Physicians are often aware of the limitations of the EIA testing and can dismiss the "in house" test as a probable false positive, resulting in unnecessary excessive testing to find other explanations for a patients’ symptoms, witholding appropriate antibiotic treatment and failure to make the necessary follow up recommendations. Sometimes the opposite is true and people receive incorrect diagnoses of acute Q fever, with over/inappropriate treatment or insufficient evaluation for other causes of their symptoms. Either way, attempts need to be made to find a more reliable "in house" test or improve the turnaround time for tests sent to the reference laboratory. There are a variety of different methods used in hospitals throughout Israel but most of them require more skilled personnel and financial resources that make them less feasible for many community hospitals.

A syndromic approach should not replace laboratory testing but may assist in the decision making process. Using this approach, we hope to reduce false positive results of EIA testing. Only patients with a relatively high likelihood for disease (as defined by our syndromic approach) would be tested. There is the inevitable risk that some cases will be missed but hopefully by improving the pre-test probability, the accuracy of the test will improve, the physicians will trust the EIA test more and respond appropriately to the results regarding treatment and follow up.

Whilst PCR testing for Q fever is highly specific[30], *C*. *burnetii* detection by PCR is most efficient in the first two weeks of acute Q fever, with reported sensitivities of 26–98%[31]. The average time from the onset of symptoms to the time of hospitalization in our study was 17 days, making PCR testing irrelevant in most of our cases.

There are several limitations to our study. As any retrospective study it may suffer from selection bias. Although a single center study, aside from the discrepancy regarding the rates of headaches, overall our findings were similar to those of another hospital in our region[18]. One must consider a possible testing bias for patients with pneumonia, a known feature of acute Q fever, that could account for our high CAP rates. We tried to mitigate this bias by comparing the infected group with a non-infected group. We have used rigid diagnostic criteria thereby reducing the chances "false positive" cases being included, but it is difficult to be completely confident of acute Q fever diagnosis without convalescent sera in all cases. We tried contacting borderline cases by phone to request repeat serology, but many were non-compliant or unreachable. We aim to continue collecting local data and work together with other institutions in Israel to build a larger database that is more representative of the situation in Israel as a whole. Limitations using IFA as the "gold standard" for diagnosis, as is done in Israel, can also be problematic because it does not allow for differentiation between past/present infection. We accounted for this by reviewing the clinical scenario surrounding each positive IFA test result.

### Conclusion

Acute Q fever is endemic in the Sharon district of Israel and yet difficult to diagnose. Unfortunately, IFA testing is limited to one reference laboratory and non-IFA tests are prone to subjective interpretation and have low specificity. We hope to improve the PPV of the EIA testing by better defining patients at higher risk of having acute Q fever. Identifying patterns of disease presentation based on geographic location may facilitate diagnosis and prevent prolonged hospitalization with unnecessary diagnostic procedures. Most importantly, timely and accurate diagnosis is more likely to lead to adequate follow up and treatment of patients at risk of developing complications of chronic Q fever. Current in-hospital diagnostic tools for Q fever are of low specificity, resulting in loss of follow-up of suspected, probable or true cases. Attempts need to be made to improve rapid diagnostic testing.

## Supporting information

S1 Data S1 Data refers to data collected regarding acute Q fever positive patients. (XLSX)

S2 Data S2 Data refers to data collected regarding non-infected control patients. (XLSX)
